# Clinical and genetic features of pediatric acute lymphoblastic leukemia in Down syndrome in the Nordic countries

**DOI:** 10.1186/1756-8722-7-32

**Published:** 2014-04-11

**Authors:** Catarina Lundin, Erik Forestier, Mette Klarskov Andersen, Kirsi Autio, Gisela Barbany, Lucia Cavelier, Irina Golovleva, Sverre Heim, Kristiina Heinonen, Randi Hovland, Johann H Johannsson, Eigil Kjeldsen, Ann Nordgren, Lars Palmqvist, Bertil Johansson

**Affiliations:** 1 Department of Clinical Genetics, University and Regional Laboratories Region Skåne, SE-221 85 Lund, Sweden; 2 Division of Clinical Genetics, Department of Laboratory Medicine, Lund University, Lund, Sweden; 3 Department of Medical Biosciences, University of Umeå, Umeå, Sweden; 4 The Cytogenetic Laboratory, The University Hospital Rigshospitalet, Copenhagen, Denmark; 5 Helsinki and Uusimaa Hospital Group, HUSLAB Laboratory of Genetics, Helsinki, Finland; 6 Department of Molecular Medicine and Surgery, Karolinska Institute, Stockholm, Sweden; 7 Department of Genetics and Pathology, Uppsala University, Uppsala, Sweden; 8 Section for Cancer Cytogenetics, Institute for Medical Informatics, The Norwegian Radium Hospital, Oslo University Hospital, and Centre for Cancer Biomedicine, Faculty of Medicine, University of Oslo, Oslo, Norway; 9 Genetic Laboratory, ISLAB, Kuopio, Finland; 10 Center of Medical Genetics and Molecular Medicine, Haukeland University Hospital, Helse-Bergen, HF, Norway; 11 Department of Clinical Genetics and Cytogenetics, University Hospital, Reykjavik, Iceland; 12 Cancer Cytogenetic Laboratory, Department of Hematology, Aarhus University Hospital, Aarhus, Denmark; 13 Department of Clinical Chemistry and Transfusion Medicine, Sahlgrenska University Hospital, Göteborg, Sweden

**Keywords:** Down syndrome, ALL, Children, Clinical features, Genetic features, Prognosis

## Abstract

**Background:**

Children with Down syndrome (DS) have an increased risk for acute lymphoblastic leukemia (ALL). Although previous studies have shown that DS-ALL differs clinically and genetically from non-DS-ALL, much remains to be elucidated as regards genetic and prognostic factors in DS-ALL.

**Methods:**

To address clinical and genetic differences between DS-ALL and non-DS-ALL and to identify prognostic factors in DS-ALL, we ascertained and reviewed all 128 pediatric DS-ALL diagnosed in the Nordic countries between 1981 and 2010. Their clinical and genetic features were compared with those of the 4,647 B-cell precursor (BCP) ALL cases diagnosed during the same time period.

**Results:**

All 128 DS-ALL were BCP ALL, comprising 2.7% of all such cases. The 5-year event-free survival (EFS) and overall survival (OS) were significantly (*P* = 0.026 and *P* = 0.003, respectively) worse for DS-ALL patients with white blood cell counts ≥50 × 10^9^/l. The age distributions varied between the DS and non-DS cases, with age peaks at 2 and 3 years, respectively; none of the DS patients had infant ALL (*P* = 0.029). The platelet counts were lower in the DS-ALL group (*P* = 0.005). Abnormal karyotypes were more common in non-DS-ALL (*P* < 0.0001), and there was a significant difference in the modal number distribution, with only 2% high hyperdiploid DS-ALL cases (*P* < 0.0001). The 5-year EFS and 5-year OS were significantly worse for DS-ALL (0.574 and 0.691, respectively) compared with non-DS-ALL (0.783 and 0.894, respectively) in the NOPHO ALL-1992/2000 protocols (*P* < 0.001).

**Conclusions:**

The present study adds further support for genetic and clinical differences between DS-ALL and non-DS-ALL.

## Background

It is well established that children with Down syndrome (DS) have an increased risk of developing acute leukemia, with a relative risk of approximately 20 and a cumulative risk of 2.1% at the age of 5 years [1,2]. Acute lymphoblastic leukemia (ALL) in DS (DS-ALL) affects 1 in 300 children with this syndrome [2,3] and several studies have shown that the outcome of DS-ALL is inferior to that of non-DS-ALL [4-6]. In fact, leukemia is one of the most common causes of death in individuals with DS, together with respiratory diseases, congenital circulatory defects, diseases of the digestive system, and Alzheimer’s disease [7]. The poor prognosis of DS-ALL has been attributed to a lower remission rate after induction therapy as well as to a higher degree of treatment-related toxicity [4,8-11].

In contrast to myeloid leukemia in DS (ML-DS), which is associated with a transient myeloproliferative disorder (TMD), DS-ALL does not display any obvious preleukemic phase [12,13]. Furthermore, specific genetic changes, such as *GATA1* mutations in TMD and ML-DS that would possibly enable early diagnosis and treatment, seem to be rare in DS-ALL [14], although deregulation of the *CRLF2* gene at Xp22.33/Yp11.32 is more prevalent in DS-ALL compared with non-DS-ALL, as are specific mutations in exon 16 of *JAK2* at 9p24.1 [15-18]. DS-ALL and non-DS-ALL have been reported to display similar median ages, gender distributions, and white blood cell (WBC) counts [13]. Thus, no clinical features, apart from the DS phenotype, at the time of diagnosis distinguish these two patient groups.

However, there are clear immunophenotypic differences between DS-ALL and non-DS-ALL, with mature B-cell ALL (Burkitt leukemia) and T-cell ALL being exceedingly rare in patients with DS; virtually all DS-ALL cases display a B-cell precursor (BCP) phenotype [11,13,19-21]. As regards acquired genetic differences, although single nucleotide polymorphism (SNP) analyses have in general identified similar microdeletions in DS-ALL and non-DS-ALL cases [16,22-25], DS-ALL and non-DS-ALL are to a large extent cytogenetically distinct – DS-ALL are rarely high hyperdiploid (51–67 chromosomes) and seldom display *MLL* rearrangements or t(9;22)(q34;q11)/*BCR*-*ABL1*[5,20,26,27]. The lack of specific ALL-associated gene fusions precludes risk group assignment and hence treatment stratification based on cytogenetics in most instances of DS-ALL.

In order to address further clinical and genetic differences between DS-ALL and non-DS-ALL and to identify prognostic factors in DS-ALL, we have ascertained and reviewed all pediatric ALL cases included in the Nordic Society of Pediatric Hematology, Oncology (NOPHO) registry 1981–2010.

## Materials and methods

### Patients

Between 1981 and 2010, a total of 128 childhood and adolescent (<18 years) DS-ALL cases were diagnosed in the Nordic countries (Denmark, Finland, Iceland, Norway, and Sweden) and registered in the NOPHO database. A subset of the 128 patients (43 cases) was included in a recently reported retrospective, international study on DS-ALL [28]. Prior to 1992, some patients may have escaped registration due to a less developed database, but since 1992 data on clinical, immunophenotypic, and genetic features of all Nordic pediatric ALL cases, at least those <15 years of age, have been entered into the NOPHO register, which hence is all-inclusive and truly population-based for this age group since that time point. All DS patients had BCP ALL, and for this reason their clinical and genetic features were compared only with those of non-DS BCP ALL cases diagnosed during the same time period (n = 4,637). The 128 DS-ALL patients were treated according to five different protocols: NOPHO ALL-1981 (n = 19), NOPHO ALL-1986 (n = 21), NOPHO ALL-1992 (n = 42), NOPHO ALL-2000 (n = 34), and NOPHO ALL-2008 (n = 12). The corresponding distribution of the 4,637 non-DS-ALL patients was NOPHO ALL-1981 (n = 563), NOPHO ALL-1986 (n = 828), NOPHO ALL-1992 (n = 1,554), NOPHO ALL-2000 (n = 1,128), and NOPHO ALL-2008 (n = 564). Details on these protocols have been reported previously [29,30]. The study was approved by the Regional Ethical Review Board at Lund University and informed consent was obtained according to the Declaration of Helsinki.

### Genetic analyses

Chromosome banding analyses were performed using standard methods in 16 cytogenetic laboratories in the Nordic countries, and all abnormal karyotypes have been centrally reviewed each year since 1996 (Sweden)/2000 (all five Nordic countries) by the Swedish and the NOPHO Leukemia Cytogenetic Study Groups. Fluorescence *in situ* hybridization (FISH) or reverse-transcription polymerase chain reaction (RT-PCR) analyses were used to screen for the translocations/gene fusions t(1;19)(q23;p13) [*TCF3/PBX1*], t(9;22)(q34;q11) [*BCR/ABL1*], and t(12;21)(p13;q22) [*ETV6/RUNX1*], whereas FISH or Southern blot analyses were used to identify 11q23/*MLL* rearrangements. These targeted analyses have been performed prospectively from 1996 in Sweden and from 2000 in the other Nordic countries, with several additional cases prior to this time having been analyzed retrospectively. During recent years, various array analyses have also been increasingly used to establish and confirm the registered karyotypes. In the NOPHO ALL-2008 protocol, FISH-based screening for intrachromosomal amplification of chromosome 21 (iAMP21) was added to the targeted analyses. Detailed karyotypic data are given in Additional file 1: Table S1.

### Statistical analysis

The PASW Statistics software for Windows (SPSS Inc., Chicago, IL) was used for all statistical analyses. The significance limit for two-sided *P* values was set to <0.05. Age, gender, WBC and platelet counts, types of event, and cytogenetic subgroups were compared within the DS-ALL group as well as between the 128 DS-ALL and the 4,637 non-DS-ALL cases using the chi square or the Mann–Whitney U tests with exact calculations of *P* values. Survival analyses were performed only on cases treated according to the NOPHO ALL-1992 and ALL-2000 protocols, partly because they were almost identical in risk stratification and treatment and partly because they comprised the majority of patients. The probabilities of event-free survival (EFS) and overall survival (OS) were calculated using the Kaplan-Meier method and subgroups were compared using the Log rank test. In the analysis of EFS, events comprised induction failure, relapse, second malignant neoplasm (SMN), and death in complete remission 1 (DCR1). In the OS analyses, death of any cause was the end point. Patients in continuous complete remission 1 were followed between 0 and 355 months (median 134 months).

## Results

### Patients

**Figure 1:**
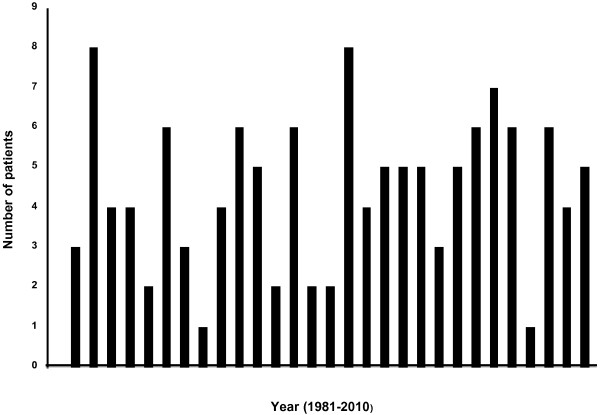
Number of DS patients diagnosed with ALL in the Nordic countries between 1981 and 2010.

The 128 DS-ALL patients comprised 2.7% of all 4,765 BCP ALL cases diagnosed and treated in the Nordic countries between 1981 and 2010, with the number of DS-ALL cases per year varying between 1 and 8 (median 5; *P* = 0.49) during this time period (Figure 1).

Within the DS-ALL cohort, there were no significant gender differences in age or WBC count distributions, whereas the platelet counts were higher in girls than in boys (median 47.0 × 10^9^/l, range 5–359, and 26.5 × 10^9^/l, range 4–493, respectively; *P* = 0.042). Age had no impact on the WBC and platelet counts.

**Figure 2:**
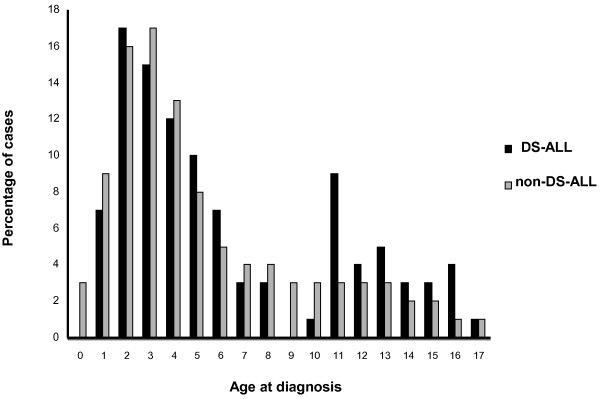
Age distribution of the DS-ALL and non-DS-ALL patients.

The DS-ALL and non-DS-ALL patients did not differ as regards sex ratio (male/female: 1.03 and 1.11, respectively) and WBC counts (median 10 × 10^9^/l, range 0.5-540, and 9 × 10^9^/l, range 0.3-1670, respectively). The median age was 4 years in both groups; however, the age distributions varied significantly (Figure 2) – the age peaks were at 2 years in DS-ALL and 3 years in non-DS-ALL, and none of the DS patients had infant ALL (*P* = 0.029). The platelet counts were lower in DS-ALL than in non-DS-ALL (median 35 × 10^9^/l; range 4–493, and 48 × 10^9^/l; range 1–878; *P* = 0.005) (Table 1).

**Table 1:** Clinical features of the 128 DS-ALL and the 4,637 non-DS-ALL patients

**Features**	**DS-ALL (%)**	**non-DS-ALL (%)**	***P *** **value**^**a**^
**Gender**			
Female	63 (49)	2195 (47)	0.67^b^
Male	65 (51)	2442 (53)	
**Age (years)**			
<1	0 (0)	154 (3.3)	**0.029**^**c**^
1-9	95 (74)	3703 (80)	
10-17	33 (26)	780 (17)	
**WBC count (×10**^**9**^**/l)**			
0-9	61 (48)	2408 (52)	0.51^c^
10-49	50 (39)	1453 (31)	
>50	17 (13)	776 (17)	
**Platelet count (×10**^**9**^**/l)**^**d**^			
<50	76 (59)	2310 (50)	**0.005**^**c**^
50-149	41 (32)	1473 (32)	
>150	6 (5)	755 (16)	
**EMD**^**e**^			
CNS	5^f^ (3.9)	125 (2.7)	NA
Mediastinal	4^f^ (3.1)	72 (1.6)	
Testis	0 (0)	15 (0.6)	

CNS, central nervous system; DS-ALL, Down syndrome-related acute lymphoblastic leukemia; EMD, extramedullary disease; NA, not analyzed due to lack of EMD data in several instances; WBC, white blood cell.

^a^Significant *P* values in bold type.

^b^Chi square test.

^c^Mann-Whitney U test.

^d^Data on platelet counts missing in 5 DS-ALL and in 99 non-DS-ALL patients.

^e^Data on CNS involvement missing in 16 non-DS-ALL patients, on mediastinal mass in one DS-ALL and in 51 non-DS-ALL patients, and on testicular involvement in 27 DS-ALL and in 990 non-DS-ALL patients.

^f^One patient had CNS involvement as well as a mediastinal mass.

### Genetic features

Informative karyotypes (normal or abnormal) at the time of diagnosis were available in 95 (74%) DS-ALL and in 3,283 (71%) non-DS-ALL cases (*P* = 0.43), with abnormal karyotypes being more common in non-DS-ALL (79%) than in DS-ALL (60%) (Table 2; *P* < 0.0001). The frequencies of normal and abnormal karyotypes in DS-ALL did not differ significantly between 1981–1995 (48% normal, 52% abnormal) and 1996–2010 (36% normal, 64% abnormal) (*P* = 0.36), whereas the proportions of karyotypic failures/missing data (1981–1995: 21% and 1996–2010: 4.7%) decreased significantly during the latter time period (*P* < 0.001). The corresponding frequencies in non-DS-ALL were 41% normal and 59% abnormal cases 1981–1995 and 14% normal and 86% abnormal cases 1996–2010 (*P* < 0.001), with the proportions of karyotypic failures/missing data being 53% for 1981–1995 and 10% for 1996–2010 (*P* < 0.001).

**Table 2:** Genetic features of the DS-ALL and the non-DS-ALL patients

**Features**	**DS-ALL (%)**	**non-DS-ALL (%)**	***P *** **value**^**a**^
**Karyotype**			
Abnormal	57 (60)	2581 (79)	**<0.0001**
Normal	38 (40)	702 (21)	
**Modal number**^**b**^			
Hypodiploidy	1 (1)	211 (6)	**<0.0001**
Pseudodiploidy	37 (39)	940 (29)	
Hyperdiploidy	17 (18)	329 (10)	
High hyperdiploidy	2 (2)	1075 (33)	
>67 chromosomes	0 (0)	26 (1)	
Normal karyotype	38 (40)	702 (21)	
***TCF3*-*PBX1***^***c***^			
Yes	1 (2.6)	70 (4.2)	1.00
No	37 (97)	1592 (96)	
***BCR*-*ABL1***^***c***^			
Yes	1 (2.1)	73 (3.7)	1.00
No	46 (98)	1923 (96)	
***MLL *** **rearrangement**^***c***^			
Yes	0 (0)	122 (7.6)	0.07
No	41 (100)	1487 (92)	
***ETV6*-*RUNX1***^***c***^			
Yes	8 (15)	568 (25)	0.11
No	46 (85)	1719 (75)	
**t(8;14)(q11;q32)**			
Yes	2 (2.1)	3 (0.09)	NA
No	93 (98)	3280 (99.9)	
**iAMP21**^**d**^			
Yes	0 (0)	6 (1.1)	NA
No	12 (100)	558 (98.9)	

DS-ALL, Down syndrome-related acute lymphoblastic leukemia; iAMP21, intrachromosomal amplification of chromosome 21; NA, not analyzed due to too few cases.

^a^Chi square test. Significant *P* values in bold type.

^b^Hypodiploidy was defined as <47 in DS and <46 in non-DS; pseudodiploidy as 47 in DS and 46 in non-DS; hyperdiploidy as 48–50 in DS and 47–50 in non-DS; and high hyperdiploidy as 51–67 chromosomes in both groups.

^c^Based only on informative cases, i.e., on which targeted molecular genetic or FISH analyses of these rearrangements had been performed.

^d^Based only on informative cases, i.e., on cases treated according to the ALL-2008 protocol and hence screened for iAMP21.

Among the DS-ALL patients, two (1.6%) had trisomy 21 mosaicism (the trisomic cells were involved in the leukemic clone), one (0.8%) had a Robertsonian translocation [der(14;21)(q10;q10)], and one (0.8%) had a complex constitutional unbalanced t(15;21;16) leading to gain of 21q; the remaining 124 (97%) patients had a classical non-mosaic trisomy 21. One patient had both DS and Klinefelter syndrome, i.e., a sex chromosome complement of XXY (Additional file 1: Table S1).

The most common acquired changes in DS-ALL were + X (14% of the 95 informative cases), deletions of 9p (13%; including some cases with microdeletions identified by array analyses), 12p (7%; including some cases with microdeletions identified by FISH and/or array analyses), 13q (6%; the majority identified by array analyses), and 1p (5%), +14/i(14)(q10) (5%), +17/i(17)(q10) (4%), and gain of an extra chromosome 21 (4%). The only recurrent translocations were t(12;21)(p13;q22), detected in 8 (15%) of the 54 cases analyzed by FISH and/or RT-PCR, and t(8;14)(q11;q32), found in two (2%) cases. The ALL-associated translocations t(1;19)(q23;p13) and t(9;22)(q34;q11) [with the P190 *BCR*/*ABL1* transcript] were found in single cases; no DS-ALL harbored an 11q23/*MLL* rearrangement or an iAMP21. The distributions of modal numbers varied significantly between DS- and non-DS-ALL cases (*P* < 0.0001), with high hyperdiploidy (51–67 chromosomes) being found in two cases only (Table 2 and Additional file 1: Table S1). Although the frequencies of t(1;19), t(9;22), and t(12;21) were lower in the DS-ALL group, they did not differ significantly from the ones observed in non-DS-ALL. Cases with t(8;14) or iAMP21 were too few to allow meaningful statistical analyses (Table 2).

### Survival of the DS-ALL patients in the NOPHO ALL-1992/2000 protocols

**Figure 3:**
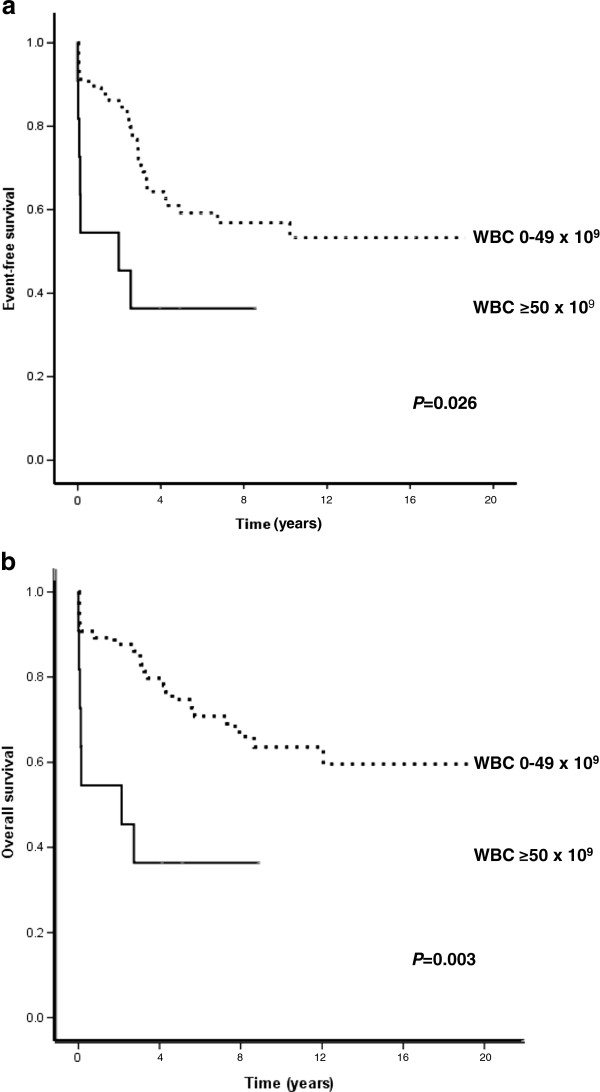
**Survival of DS-ALL patients treated according to the NOPHO ALL-1992 and ALL-2000 protocols in relation to WBC counts <50 × 10**^**9**^**/l *vs. *≥50 × 10**^**9**^**/l. (a)** Event-free survival. **(b)** Overall survival.

Of the 128 DS patients, 42 and 34 were treated according to the ALL-1992 and ALL-2000 protocols, respectively. There were no significant differences (*P* = 0.10) in 5-year EFS of the DS-ALL patients between these treatment regimens [pEFS 0.50 (standard error 0.077) *vs.* pEFS 0.67 (0.082)] or as regards 5-year OS (*P* = 0.31) [pOS 0.595 (0.076) *vs.* pOS 0.735 (0.085)]. Thus, these two protocols were combined in the subsequent survival analyses (Table 3). Within the DS-ALL cohort, gender, age and karyotype (abnormal *vs.* normal) had no impact on EFS or OS. The low number of ALL-associated translocations in DS-ALL precluded detailed analyses of their prognostic impact. However, it may be noteworthy that seven of the eight t(12;21)-positive DS-ALL patients remain in complete remission (Additional file 1: Table S1). There was a significant difference in 5-year EFS (*P* = 0.026) and in 5-year OS (*P* = 0.003) between DS-ALL patients with WBC counts below and above 50 × 10^9^/l (Figure 3; Table 3).

**Table 3:** Survival of the DS-ALL patients treated according to the ALL-1992/2000 protocols in relation to clinical and genetic features

**Features**	**5-year EFS (SE)**	***P *** **value**^**a**^	**5-year OS (SE)**	***P *** **value**^**a**^
**Gender**				
Female	0.619 (0.080)	0.46	0.693 (0.078)	0.50
Male	0.534 (0.080)		0.621 (0.081)	
**Age (years)**				
0-4	0.479 (0.106)	0.88	0.632 (0.091)	0.77
≥5	0.558 (0.076)		0.668 (0.073)	
**WBC count (×10**^**9**^**/l)**				
0-9	0.574 (0.086)	0.78	0.752 (0.076)	0.36
≥10	0.574 (0.077)		0.644 (0.074)	
0-49	0.610 (0.061)	**0.026**	0.747 (0.055)	**0.003**
≥50	0.364 (0.145)		0.364 (0.145)	
**Karyotype**				
Abnormal	0.558 (0.076)	0.86	0.668 (0.073)	0.92
Normal	0.468 (0.116)		0.661 (0.107)	

DS-ALL, Down syndrome-related acute lymphoblastic leukemia; EFS, event-free survival; OS, overall survival; SE, standard error; WBC, white blood cell.

^a^Significant *P* values in bold type.

### Survival differences between the DS-ALL and non-DS-ALL patients in the NOPHO ALL-1992/2000 protocols

Among the 4,637 non-DS-ALL patients, 1,554 and 1,128 were treated according to ALL-1992 and ALL-2000, respectively. There were no significant differences (*P* = 0.76) in 5-year EFS of the non-DS-ALL patients between these two protocols [pEFS 0.782 (0.010) *vs.* pEFS 0.785 (0.013)] or in 5-year OS (*P* = 0.51) [pOS 0.889 (0.008) *vs.* pOS 0.902 (0.009)]. These two protocols could hence be combined when comparing DS-ALL and non-DS-ALL.

The types of event varied significantly (*P* = 0.005) between the DS-ALL and non-DS-ALL cases, with induction failure being more common in DS-ALL and SMN being observed only among non-DS-ALL patients (Table 4). Nine DS-ALL patients had induction failure, of which 6 died due to toxicity (Table 4). The frequencies of induction failure did not differ between cases with WBC counts below or above 50 × 10^9^/l (data not shown).

**Table 4:** **Significant difference (*P*** **= 0.005)**^**a **^**in types of event in DS-ALL and non-DS-ALL patients treated according to the ALL-1992/2000 protocols**

**Events**	**DS-ALL (% of events)**	**non-DS-ALL (% of events)**
Relapse	23 (66)	507 (80)
Induction failure	9^b^ (26)	47 (7.4)
DCR1	3 (8.6)	48 (7.6)
SMN	0 (0)	32 (5.0)

DS-ALL, Down syndrome-related acute lymphoblastic leukemia; DCR1, death in complete remission 1; SMN, second malignant neoplasm.

^a^Mann-Whitney U test.

^b^Six of the 9 patients with induction failure died due to toxicity.

**Figure 4:**
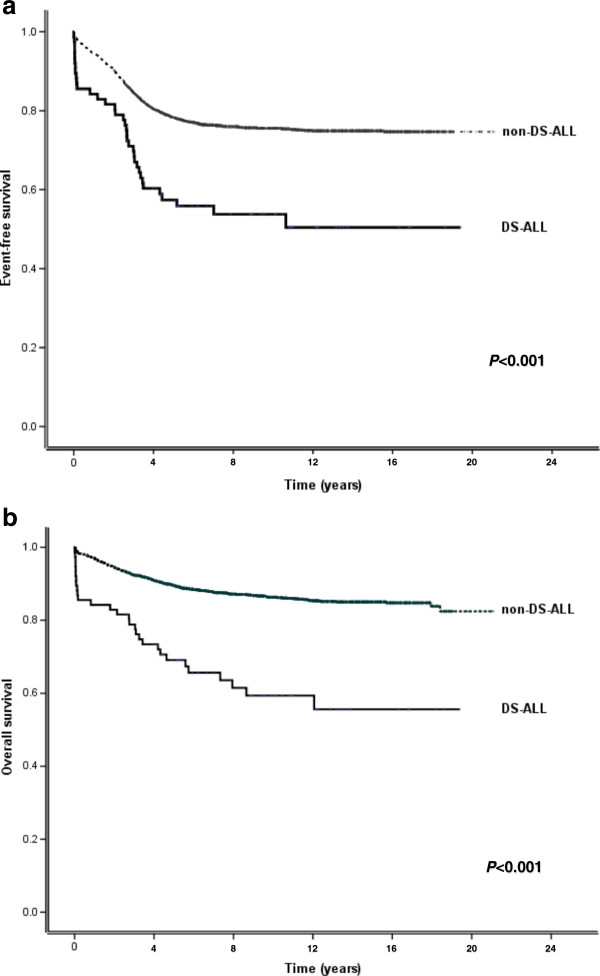
**Event-free survival (EFS) and overall survival (OS) of DS-ALL and non-DS-ALL patients in the NOPHO ALL-1992 and ALL-2000 combined. (a)** EFS in ALL-1992/2000 **(b)** OS in ALL-1992/2000.

When analysing the two treatment protocols together, both 5-year EFS and 5-year OS were significantly inferior in the DS-ALL cohort (Figure 4; Table 5).

**Table 5:** Comparison of survival data between the DS-ALL and non-DS-ALL patients treated according to the ALL-1992/2000 protocols

**Protocol**	**5-year EFS (SE)**	***P *** **value**^**a**^	**5-year OS (SE)**	***P *** **value**^**a**^
DS-ALL	0.574 (0.057)	**<0.001**	0.691 (0.054)	**<0.001**
non-DS-ALL	0.783 (0.008)		0.894 (0.006)	

DS-ALL, Down syndrome-related acute lymphoblastic leukemia; EFS, event-free survival; OS, overall survival; SE, standard error.

^a^Significant *P* values in bold type.

## Discussion

The basic clinical features of this population-based DS-ALL patient cohort from the Nordic countries (Table 1) agree very well with previous studies on DS-ALL. For example, there were no infants, no case had mature B-cell leukemia or T-cell ALL, there were no differences between the DS-ALL and the non-DS-ALL patients in sex ratio, WBC counts, and median age at diagnosis, and the platelet counts were lower in DS-ALL [4,8,11,13,20,26]. Thus, the present series is clearly representative of DS-ALL.

Since our study includes ALL cases diagnosed during the last three decades, it provides data pertinent to possible time-related aspects of diagnostic routines. We had anticipated that there could have been a bias as regards reporting DS-ALL cases to the registry during its early years, perhaps because some centres may have been less inclined to give intensive treatment to children with DS and/or to include them in the database. However, this was clearly not the case. The incidence of DS-ALL has been quite constant throughout the years, with no significant variation (Figure 1). Furthermore, we have no evidence that DS-ALL cases have been less well cytogenetically characterized through the years. In contrast, although karyotypic failures/missing data for DS-ALL were much more common 1981–1995 (21%) than between 1996 and 2010 (4.7%), the corresponding frequencies in non-DS-ALL were even higher (53% and 10%, respectively). This notwithstanding, whereas the proportion of abnormal karyotypes in DS-ALL has not increased greatly during the last few years, there has been a significant increase in the frequency of abnormal karyotypes in non-DS-ALL. We do not believe that this is due to technical advances in cytogenetic analyses only, which seem to have benefited non-DS-ALL more than DS-ALL, but rather to true biological differences.

As alluded to above, DS-ALL significantly less often displayed an abnormal karyotype (60%) compared with non-DS-ALL (79%) (Table 2). A similarly low frequency of clonal abnormalities (54%) was also noted in a previous collaborative, international cytogenetic study on acute leukemia in DS patients [26]. One reason for the higher proportion of normal karyotypes in DS-ALL could be that they more often harbor cytogenetically cryptic changes. In fact, deregulation of the *CRLF2* gene*,* either through deletions of Xp22.33/Yp11.32 or by translocations between these regions and the immunoglobulin heavy chain locus (IGH@) at 14q32.33, both undetectable by conventional chromosome banding analysis, is substantially more common in DS-ALL than in non-DS-ALL [17,18]. Furthermore, a “normal” karyotype in DS-ALL has an additional chromosome 21. Perhaps fewer additional, acquired aberrations are needed to establish the leukemia because of the presence of the “preleukaemic” trisomy 21, akin to what has been suggested for ML-DS, in which *GATA1* mutations are only efficient in the context of +21c [31]. In fact, it has been demonstrated that trisomy 21 as such perturbs fetal lymphopoiesis [32].

In contrast to the study by Forestier *et al.*[26], in which it was reported that 11% of DS-ALL cases are high hyperdiploid, only 2% of the Nordic cases had a modal number between 51 and 67 chromosomes [27] (Table 2). The reason(s) for this pronounced difference is unclear. Although high hyperdiploid cells are notoriously difficult to culture *in vitro* and many cases may escape detection, masquerading as karyotypically normal or cytogenetic failures [33,34], we deem it unlikely that the low frequency of such cases in our cohort is due to technical issues. First, the proportion of high hyperdiploid cases among the non-DS-ALL patients (33%) is on a par with other study groups [34]. Second, DNA index analyses of pediatric leukemias are performed in clinical routine, according to the NOPHO protocols, and they should have identified high hyperdiploid cases that were cytogenetically missed. Third, most laboratories in the Nordic countries use interphase FISH analyses to identify t(12;21) and all use FISH to screen for iAMP21. Since the vast majority of high hyperdiploid ALL have tetrasomy 21 [35], such analyses would also identify extra copies of chromosome 21 and hence suggest the presence of high hyperdiploidy. Furthermore, Heerema *et al*. [36] reported only four DS patients (0.8%) among 480 high hyperdiploid ALL cases. Thus, we believe that there may have been a bias in reporting high hyperdiploid DS-ALL cases in Forestier *et al.*[26].

Other ALL-associated abnormalities that were less common, but not significantly so, in DS-ALL were t(1;19), t(9;22), 11q23/*MLL* rearrangements, and t(12;21) (Table 2). However, the frequencies of t(1;19), t(9;22), and t(12;21) were slightly higher in this study compared with those reported in Forestier *et al.*[26]. This most likely reflects that targeted analyses for these abnormalities were performed in a larger proportion of Nordic DS-ALL cases. As regards iAMP21, this aberration has been actively screened for only in the ongoing NOPHO ALL-2008 protocol. So far, none of 12 DS-ALL cases treated according to this protocol has harbored an iAMP21 whereas it has been detected in 1% of non-DS-ALL cases (Table 2). The low frequency of iAMP21 in DS-ALL agrees well with data from UK MRC ALL97 [37]. The only structural abnormality that seems to be more common in DS-ALL is t(8;14)(q11;q32) (Table 2), something that has been noted also in several previous studies [38-40]. Why most ALL-associated abnormalities are less frequent or, as regards t(8;14), more common in DS-ALL remain enigmatic.

The distribution of types of event differed significantly between the DS-ALL and non-DS-ALL patients (Table 4). The fact that induction failure was more common in the former group was not surprising; this has been repeatedly observed [4,11,41]. However, the lack of secondary malignant neoplasms (SMN) after treatment for DS-ALL is noteworthy – none of the 128 DS-ALL patients in the Nordic countries has developed SMN. Although not stressed in previous studies, this seems to be the case also in several other treatment trials, such as those from the Berlin-Frankfurt-Münster (BFM), Children's Cancer Group (CCG), and Italian Association of Pediatric Hematology and Oncology (AIEOP) groups [10,11,41,42]. It is well known that individuals with DS in general have a lower incidence of solid tumors than non-DS-individuals [2], with possible explanations including gains of tumor suppressor genes on chromosome 21, impaired angiogenesis, and accelerated cell ageing [43]. Whether such mechanisms could be involved in preventing SMN in DS patients is unclear, not least considering that most SMN are not solid tumors but rather myelodysplastic syndromes and acute myeloid leukemia, with the latter being clearly overrepresented in DS as a *de novo* disease [2].

The only factor associated with survival in DS-ALL was the WBC count. Patients with a WBC count ≥50 × 10^9^/l had significantly inferior 5-year EFS and 5-year OS compared with those with lower counts (Table 3; Figure 3). Although not surprising – a high WBC count is a well-established risk factor in pediatric non-DS-ALL [30] – this has not been clearly demonstrated in DS-ALL previously.

Data on improved outcome for DS-ALL have emerged during the last few years from some treatment trials [41,42,44], suggesting that induction and/or maintenance therapy has improved for this patient cohort. However, in the present study, both EFS and OS were still inferior for the DS-ALL cohort in the combined ALL-1992/2000 protocols (Figure 4; Table 5). Whether this depends on insufficient treatment intensity [45], clinical decision making for relapsed DS-ALL, or on treatment- and/or syndrome-related comorbidity [46] remains to be evaluated in detail.

## Competing interests

The authors declare that they have no competing interests.

## Authors’ contributions

CL and BJ designed the study, analyzed data, and wrote the article; EF provided critical comments on the design of the study, analysis, and of the interpretation of data; all authors supplied data, critically revised, and gave final approval of the article.

## Supplementary Material

Additional file 1 Clinical and karyotypic data on the 128 DS-ALL patients. Click here for file

## References

[B1] Krivit W Good RA Simultaneous occurrence of mongolism and leukemia: report of a nationwide survey AMA J Dis Child 1957 94 289 293 13457660 10.1001/archpedi.1957.04030040075012

[B2] Hasle H Clemmensen IH Mikkelsen M Risks of leukaemia and solid tumours in individuals with Down's syndrome Lancet 2000 355 165 169 10675114 10.1016/S0140-6736(99)05264-2

[B3] Lange B The management of neoplastic disorders of haematopoiesis in children with Down's syndrome Br J Haematol 2000 10 512 524 10997960 10.1046/j.1365-2141.2000.02027.x

[B4] Zeller B Gustafsson G Forestier E Abrahamsson J Clausen N Heldrup J Hovi L Jonmundsson G Lie SO Glomstein A Hasle H Acute leukaemia in children with Down syndrome: a population-based Nordic study Br J Haematol 2005 128 797 804 15755283 10.1111/j.1365-2141.2005.05398.x

[B5] Maloney KW Carroll WL Carroll AJ Devidas M Borowitz MJ Martin PL Pullen J Whitlock JA Willman CL Winick NJ Camitta BM Hunger SP Down syndrome childhood acute lymphoblastic leukemia has a unique spectrum of sentinel cytogenetic lesions that influences treatment outcome: a report from the Children's Oncology Group Blood 2010 116 1045 1050 20442364 10.1182/blood-2009-07-235291 PMC2938126

[B6] Seewald L Taub JW Maloney KW McCabe ER Acute leukemias in children with Down syndrome Mol Genet Metab 2012 107 25 30 22867885 10.1016/j.ymgme.2012.07.011

[B7] Day SM Strauss DJ Shavelle RM Reynolds RJ Mortality and causes of death in persons with Down syndrome in California Dev Med Child Neurol 2005 47 171 176 15739721 10.1017/s0012162205000319

[B8] Robison LL Nesbit ME Jr Sather HN Level C Shahidi N Kennedy A Hammond D Down syndrome and acute leukemia in children: a 10-year retrospective survey from Childrens Cancer Study Group J Pediatr 1984 105 235 242 6235337 10.1016/s0022-3476(84)80119-5

[B9] Levitt GA Stiller CA Chessells JM Prognosis of Down's syndrome with acute leukaemia Arch Dis Child 1990 65 212 216 2138446 10.1136/adc.65.2.212 PMC1792207

[B10] Dördelmann M Schrappe M Reiter A Zimmermann M Graf N Schott G Lampert F Harbott J Niemeyer C Ritter J Dörffel W Nessler G Kühl J Riehm H Down's syndrome in childhood acute lymphoblastic leukemia: clinical characteristics and treatment outcome in four consecutive BFM trials Leukemia 1998 12 645 651 9593260 10.1038/sj.leu.2400989

[B11] Whitlock JA Sather HN Gaynon P Robison LL Wells RJ Trigg M Heerema NA Bhatia S Clinical characteristics and outcome of children with Down syndrome and acute lymphoblastic leukemia: a Children's Cancer Group study Blood 2005 106 4043 4049 16109782 10.1182/blood-2003-10-3446

[B12] Henry E Walker D Wiedmeier SE Christensen RD Hematological abnormalities during the first week of life among neonates with Down syndrome: data from a multihospital healthcare system Am J Med Genet A 2007 143 42 50 17163522 10.1002/ajmg.a.31442

[B13] Maloney KW Acute lymphoblastic leukaemia in children with Down syndrome: an updated review Br J Haematol 2011 155 420 425 21933171 10.1111/j.1365-2141.2011.08846.x

[B14] Roy A Roberts I Vyas P Biology and management of transient abnormal myelopoiesis (TAM) in children with Down syndrome Semin Fetal Neonatal Med 2012 17 196 201 22421527 10.1016/j.siny.2012.02.010

[B15] Bercovich D Ganmore I Scott LM Wainreb G Birger Y Elimelech A Shochat C Cazzaniga G Biondi A Basso G Cario G Schrappe M Stanulla M Strehl S Haas OA Mann G Binder V Borkhardt A Kempski H Trka J Bielorei B Avigad S Stark B Smith O Dastugue N Bourquin JP Tal NB Green AR Izraeli S Mutations of *JAK2* in acute lymphoblastic leukaemias associated with Down's syndrome Lancet 2008 372 1484 1492 18805579 10.1016/S0140-6736(08)61341-0

[B16] Kearney L Gonzalez De Castro D Yeung J Procter J Horsley SW Eguchi-Ishimae M Bateman CM Anderson K Chaplin T Young BD Harrison CJ Kempski H So CWE Ford AM Greaves M Specific *JAK2* mutation (JAK2R683) and multiple gene deletions in Down syndrome acute lymphoblastic leukaemia Blood 2009 113 646 648 18927438 10.1182/blood-2008-08-170928

[B17] Mullighan CG Collins-Underwood JR Phillips LAA Loudin MG Liu W Zhang J Ma J Coustan-Smith E Harvey RC Willman CL Mikhail FM Meyer J Carroll AJ Williams RT Cheng J Heerema NA Basso G Pession A Pui C-H Raimondi SC Hunger SP Downing JR Carroll WL Rabin KR Rearrangement of *CRLF2* in B-progenitor and Down syndrome-associated acute lymphoblastic leukemia Nat Genet 2009 41 1243 1246 19838194 10.1038/ng.469 PMC2783810

[B18] Russell LJ Capasso M Vater I Akasaka T Bernard OA Calasanz MJ Chandrasekaran T Chapiro E Gesk S Griffiths M Guttery DS Haferlach C Harder L Heidenreich O Irving J Kearney L Nguyen-Khac F Machado L Minto L Majid A Moorman AV Morrison H Rand V Strefford JC Schwab C Tönnies H Dyer MJS Siebert R Harrison CJ Deregulated expression of cytokine receptor gene, *CRLF2*, is involved in lymphoid transformation in B-cell precursor acute lymphoblastic leukemia Blood 2009 114 2688 2698 19641190 10.1182/blood-2009-03-208397

[B19] Ragab AH Abdel-Mageed A Shuster JJ Frankel LS Pullen J van Eys J Sullivan MP Boyett J Borowitz M Crist WM Clinical characteristics and treatment outcome of children with acute lymphocytic leukemia and Down's syndrome. A Pediatric Oncology Group study Cancer 1991 67 1057 1063 1825025 10.1002/1097-0142(19910215)67:4<1057::aid-cncr2820670432>3.0.co;2-k

[B20] Pui C-H Raimondi SC Borowitz MJ Land VJ Behm FG Pullen DJ Hancock ML Shuster JJ Steuber CP Crist WM Civin CI Carroll AJ Immunophenotypes and karyotypes of leukemic cells in children with Down syndrome and acute lymphoblastic leukemia J Clin Oncol 1993 11 1361 1367 8315434 10.1200/JCO.1993.11.7.1361

[B21] Chessells JM Harrison G Richards SM Bailey CC Hill FG Gibson BE Hann IM Down's syndrome and acute lymphoblastic leukaemia: clinical features and response to treatment Arch Dis Child 2001 85 321 325 11567943 10.1136/adc.85.4.321 PMC1718934

[B22] Kawamata N Ogawa S Zimmermann M Kato M Sanada M Hemminki K Yamatomo G Nannya Y Koehler R Flohr T Miller CW Harbott J Ludwig W-D Stanulla M Schrappe M Bartram CR Koeffler HP Molecular allelokaryotyping of pediatric acute lymphoblastic leukemias by high-resolution single nucleotide polymorphism oligonucleotide genomic microarray Blood 2008 111 776 784 17890455 10.1182/blood-2007-05-088310 PMC2200831

[B23] Hertzberg L Vendramini E Ganmore I Cazzaniga G Schmitz M Chalker J Shiloh R Iacobucci I Shochat C Zeligson S Cario G Stanulla M Strehl S Russell LJ Harrison CJ Bornhauser B Yoda A Rechavi G Bercovich D Borkhardt A Kempski H te Kronnie G Bourquin J-P Domany E Izraeli S Down syndrome acute lymphoblastic leukemia, a highly heterogeneous disease in which aberrant expression of *CRLF2* is associated with mutated *JAK2*: a report from the International BFM Study Group Blood 2010 115 1006 1017 19965641 10.1182/blood-2009-08-235408

[B24] Loudin MG Wang J Eastwood Leung HC Gurusiddappa S Meyer J Condos G Morrison D Tsimelzon A Devidas M Heerema NA Carroll AJ Plon SE Hunger SP Basso G Pession A Bhojwani D Carroll WL Rabin KR Genomic profiling in Down syndrome acute lymphoblastic leukemia identifies histone gene deletions associated with altered methylation profiles Leukemia 2011 25 1555 1563 21647151 10.1038/leu.2011.128 PMC4107887

[B25] Lundin C Hjorth L Behrendtz M Nordgren A Palmqvist L Andersen MK Biloglav A Forestier E Paulsson K Johansson B High frequency of *BTG1* deletions in acute lymphoblastic leukemia in children with Down syndrome Genes Chromosomes Cancer 2012 51 196 206 22072402 10.1002/gcc.20944

[B26] Forestier E Izraeli S Beverloo B Haas O Pession A Michalová K Stark B Harrison CJ Teigler-Schlegel A Johansson B Cytogenetic features of acute lymphoblastic and myeloid leukemias in pediatric patients with Down syndrome: an iBFM-SG study Blood 2008 111 1575 1583 17971484 10.1182/blood-2007-09-114231

[B27] Paulsson K Forestier E Andersen MK Autio K Barbany G Borgström G Cavelier L Golovleva I Heim S Heinonen K Hovland R Johannsson JH Kjeldsen E Nordgren A Palmqvist L Johansson B High modal number and triple trisomies are highly correlated favorable factors in childhood B-cell precursor high hyperdiploid acute lymphoblastic leukemia treated according to the NOPHO ALL 1992/2000 protocols Haematologica 2013 98 1424 1432 23645689 10.3324/haematol.2013.085852 PMC3762100

[B28] Buitenkamp TD Izraeli S Zimmermann M Forestier E Heerema NA van den Heuvel-Eibrink MM Pieters R Korbijn CM Silverman LB Schmiegelow K Liang DC Horibe K Arico M Biondi A Basso G Rabin KR Schrappe M Cario G Mann G Morak M Panzer-Grümayer R Mondelaers V Lammens T Cavé H Stark B Ganmore I Moorman AV Vora A Hunger SP Pui CH Acute lymphoblastic leukemia in children with Down syndrome: a retrospective analysis from the Ponte di Legno study group Blood 2014 123 70 77 24222333 10.1182/blood-2013-06-509463 PMC3879907

[B29] Gustafsson G Schmiegelow K Forestier E Clausen N Glomstein A Jonmundsson G Mellander L Makipernaa A Nygaard R Saarinen-Pihkala UM Improving outcome through two decades in childhood ALL in the Nordic countries: the impact of high-dose methotrexate in the reduction of CNS irradiation Leukemia 2000 14 2267 2275 11187918 10.1038/sj.leu.2401961

[B30] Schmiegelow K Forestier E Hellebostad M Heyman M Kristinsson J Söderhäll S Taskinen M Long-term results of NOPHO ALL-92 and ALL-2000 studies of childhood acute lymphoblastic leukemia Leukemia 2010 24 345 354 20010622 10.1038/leu.2009.251

[B31] Malinge S Izraeli S Crispino JD Insights into the manifestations, outcomes, and mechanisms of leukemogenesis in Down syndrome Blood 2009 113 2619 2628 19139078 10.1182/blood-2008-11-163501 PMC2661853

[B32] Roy A Cowan G Mead AJ Filippi S Bohn G Chaidos A Tunstall O Chan JK Choolani M Bennett P Kumar S Atkinson D Wyatt-Ashmead J Hu M Stumpf MP Goudevenou K O'Connor D Chou ST Weiss MJ Karadimitris A Jacobsen SE Vyas P Roberts I Perturbation of fetal liver hematopoietic stem and progenitor cell development by trisomy 21 Proc Natl Acad Sci U S A 2012 109 17579 17584 23045701 10.1073/pnas.1211405109 PMC3491522

[B33] Harrison CJ Moorman AV Barber KE Broadfield ZJ Cheung KL Harris RL Jalali GR Robinson HM Strefford JC Stewart A Wright S Griffiths M Ross FM Harewood H Martineau M Interphase molecular cytogenetic screening for chromosomal abnormalities of prognostic significance in childhood acute lymphoblastic leukaemia: a UK Cancer Cytogenetics group study Br J Haematol 2005 129 520 530 15877734 10.1111/j.1365-2141.2005.05497.x

[B34] Paulsson K Johansson B High hyperdiploid childhood acute lymphoblastic leukemia Genes Chromosomes Cancer 2009 48 637 660 19415723 10.1002/gcc.20671

[B35] Paulsson K Forestier E Lilljebjörn H Heldrup J Behrendtz M Young BD Johansson B Genetic landscape of high hyperdiploid childhood acute lymphoblastic leukemia Proc Natl Acad Sci U S A 2010 107 21719 21724 21098271 10.1073/pnas.1006981107 PMC3003126

[B36] Heerema NA Sather HN Sensel MG Zhang T Hutchinson RJ Nachman JB Lange BJ Steinherz PG Bostrom BC Reaman GH Gaynon PS Uckun FM Prognostic impact of trisomies of chromosomes 10, 17, and 5 among children with acute lymphoblastic leukemia and high hyperdiploidy (>50 chromosomes) J Clin Oncol 2000 18 1876 1887 10784628 10.1200/JCO.2000.18.9.1876

[B37] Moorman AV Richards SM Robinson HM Strefford JC Gibson BES Kinsey SE Eden TOB Vora AJ Mitchell CD Harrison CJ Prognosis of children with acute lymphoblastic leukemia (ALL) and intrachromosomal amplification of chromosome 21 (iAMP21) Blood 2007 109 2327 2330 17095619 10.1182/blood-2006-08-040436

[B38] Secker-Walker LM Hawkins JM Prentice HG Mackie PH Heerema NA Provisor AJ Two Down syndrome patients with an acquired translocation, t(8;14)(q11;q32), in early B-lineage acute lymphoblastic leukemia Cancer Genet Cytogenet 1993 70 148 150 8242599 10.1016/0165-4608(93)90189-s

[B39] Lundin C Heldrup J Ahlgren T Olofsson T Johansson B B-cell precursor t(8;14)(q11;q32)-positive acute lymphoblastic leukemia in children is strongly associated with Down syndrome or with a concomitant Philadelphia chromosome Eur J Haematol 2009 82 46 53 19067745 10.1111/j.1600-0609.2008.01166.x

[B40] Messinger YH Higgins RR Devidas M Hunger SP Carroll AJ Heerema NA Pediatric acute lymphoblastic leukemia with a t(8;14)(q11.2;q32): B-cell disease with a high proportion of Down syndrome: a Children's Oncology Group study Cancer Genet 2012 205 453 458 22939398 10.1016/j.cancergen.2012.07.016 PMC3432955

[B41] Arico M Ziino O Valsecchi MG Cazzaniga G Baronci C Messina C Pession A Santoro N Basso G Conter V Acute lymphoblastic leukemia and Down syndrome: presenting features and treatment outcome in the experience of the Italian Association of Pediatric Hematology and Oncology (AIEOP) Cancer 2008 113 515 521 18521927 10.1002/cncr.23587

[B42] Meyr F Escherich G Mann G Klingebiel T Kulozik A Rossig C Schrappe M Henze G von Stackelberg A Hitzler J Outcomes of treatment for relapsed acute lymphoblastic leukaemia in children with Down syndrome Br J Haematol 2013 162 98 106 23594030 10.1111/bjh.12348

[B43] Nižetić D Groet J Tumorigenesis in Down’s syndrome: big lessons from a small chromosome Nat Rev Cancer 2012 12 721 732 22996602 10.1038/nrc3355

[B44] Shah N Al-Ahmari A Al-Yamani A Dupuis L Stephens D Hitzler J Outcome and toxicity of chemotherapy for acute lymphoblastic leukemia in children with Down syndrome Pediatr Blood Cancer 2009 52 14 19 18802938 10.1002/pbc.21737

[B45] Bohnstedt C Levinsen M Rosthøj S Zeller B Taskinen M Hafsteinsdottir S Björgvinsdóttir H Heyman M Schmiegelow K Physicians compliance during maintenance therapy in children with Down syndrome and acute lymphoblastic leukemia Leukemia 2013 27 866 870 23138181 10.1038/leu.2012.325

[B46] Patrick K Wade R Goulden N Rowntree C Hough R Moorman AV Mitchell CD Vora A Outcome of Down syndrome associated acute lymphoblastic leukaemia treated on a contemporary protocol Br J Haematolin press 10.1111/bjh.12739 24428704

