# Supplementary material for: Clinical and genetic features of pediatric acute lymphoblastic leukemia in Down syndrome in the Nordic countries
Source: J Hematol Oncol. 2014 Apr 11;7:32. doi: 10.1186/1756-8722-7-32 (PMC4022076; doi:10.1186/1756-8722-7-32)
Supplement: Additional file 1 — Clinical and karyotypic data on the 128 DS-ALL patients. [file 1756-8722-7-32-S1.doc]

**Additional file 1: Table S1.** Clinical and karyotypic data on the 128 DS-ALL patients

| **Case**  **No.^a^** | **Sex/**  **age (years)** | **WBC**  **(x10^9^/l)** | **Plts**  **(x10^9^/l)** | **Survival**  **(months)** | **Karyotype** |
| --- | --- | --- | --- | --- | --- |
| 1 | M/2 | 15.0 | 14 | 12 | Missing |
| 2 | M/11 | 11.0 | 29 | 206+ | Missing |
| 3 | M/11 | 5.0 | 110 | 94 | Missing |
| 4 | M/3 | 14.0 | 20 | 4 | Missing |
| 5 | M/2 | 7.0 | 26 | 82 | 47,XY,+21c |
| 6 | M/2 | 36.0 | 32 | 329 | Missing |
| 7 | M/4 | 5.0 | 21 | 324+ | Missing |
| 8 | F/1 | 300 | NK | 0 | Missing |
| 9 | M/3 | 3.0 | 55 | 258 | Missing |
| 10 | M/5 | 3.0 | 24 | 227+ | Missing |
| 11 | F/1 | 7.0 | 5 | 290+ | Missing |
| 12 | M/13 | 9.0 | 18 | 109 | Missing |
| 13 | F/2 | 53.0 | 61 | 1 | Missing |
| 14 | M/7 | 31.0 | 12 | 67 | Missing |
| 15 | F/4 | NK | NK | 0 | Missing |
| 16 | M/3 | 5.0 | 12 | 100 | Missing |
| 17 | F/2 | 20.0 | 115 | 280+ | Missing |
| 18 | M/2 | 12.0 | 4 | 0 | Missing |
| 19 | F/5 | 31.0 | 10 | 2 | Missing |
| 20 | F/2 | 4.0 | 34 | 25 | Missing |
| 21 | F/16 | 21.0 | 26 | 165+ | 47,XX,+21c |
| 22 | F/2 | 2.0 | 35 | 146+ | 47,XX,i(9)(q10),i(14)(q10),+21c |
| 23 | M/2 | 21.0 | 8 | 280+ | Missing |
| 24 | M/3 | 2.0 | 24 | 19 | 47,XY,+21c |
| 25 | M/11 | 5.0 | 493 | 270+ | Missing |
| 26 | F/5 | 6.0 | 47 | 147+ | 47,XX,+21c |
| 27 | F/2 | 17.0 | 83 | 1 | 47,XX,del(19)(p13),+21c |
| 28 | M/14 | 10.0 | 46 | 271+ | 47,XY,+21c |
| 29 | M/1 | 21.0 | NK | 273+ | 47,XY,+21c |
| 30 | M/3 | 5.0 | 20 | 50 | Missing |
| 31 | F/1 | 5.0 | 19 | 255+ | 47,XX,t(3;19),+21c |
| 32 | M/4 | 4.0 | 39 | 115+ | 47,XY,r(?12),del(21),+21c |
| 33 | F/14 | 97.0 | 60 | 113+ | 47,XX,t(8;14)(q11;q32),+21c |
| 34 | F/11 | 6.0 | 10 | 249+ | 47,XX,+21c |
| 35 | F/12 | 5.0 | NK | 197+ | 47,XX,+21c |
| 36^b^ | F/2 | 15.0 | 13 | 187+ | 48,XX,+del(X)(p22p22),+21c [partly based on SNP array results] |
| 37 | F/15 | 1.0 | 150 | 100+ | 48,XX,add(14)(q?),+21c,+mar |
| 38 | M/4 | 6.0 | 111 | 12 | 47,XY,+21c |
| 39 | F/2 | 7.0 | 120 | 101+ | 47,XX,del(17)(p1?2),+21c |
| 40 | M/4 | 78.0 | 18 | 26 | 48,XY,+21c,+mar |
| 41 | F/8 | 104 | 58 | 32 | 49,XX,+X,-16,i(17)(q10),-20,+21c,del(22)(q?),inc |
| 42 | M/2 | 7.6 | 11 | 202+ | 48,XY,del(6)(q?),+14,+21c |
| 43^b^ | M/16 | 13.0 | 11 | 2 | 47,XY,t(8;14)(q11;q32),inv(12)(q13q24),der(14)t(8;14),+21c |
| 44 | M/7 | 26.9 | 37 | 218+ | 47,XY,+21c |
| 45 | F/12 | 16.9 | 21 | 224+ | Missing |
| 46 | F/2 | 22.9 | 38 | 233+ | Missing |
| 47 | M/2 | 8.7 | 100 | 39 | 47,XY,+21c |
| 48 | M/13 | 540 | 36 | 0 | Missing |
| 49 | M/3 | 6.0 | 16 | 1 | Missing |
| 50 | F/1 | 6.6 | 32 | 196+ | 48,XX,+8,+21c/48,idem,del(11)(q22) |
| 51 | M/2 | 2.6 | 91 | 37 | 47,XY,+21c |
| 52 | M/5 | 18.1 | 22 | 203+ | 47,XY,+21c |
| 53 | F/3 | 29.8 | 101 | 145 | 48-50,XX,+X,+21c,inc |
| 54 | F/6 | 19.5 | 42 | 55 | 47,XX,der(16),+21c |
| 55 | F/4 | 14.8 | 18 | 52 | 47,XX,+21c |
| 56 | F/3 | 1.9 | 192 | 1 | Missing |
| 57 | F/16 | 5.4 | 74 | 37 | 47,XX,+21c |
| 58 | M/5 | 5.1 | 74 | 69 | 47,XY,+21c |
| 59^b^ | M/6 | 25.0 | 10 | 165+ | 47,XY,del(3)(p21p21),del(9)(p13p13),del(12)(p12p13),del(12)(q21q21),t(12;21)(p13;q22),+21c  [partly based on FISH and SNP array results] |
| 60 | M/7 | 28.0 | 25 | 104 | 47,XY,+21c |
| 61 | F/2 | 61.0 | 44 | 0 | 47,XX,+21c |
| 62 | F/2 | 5.0 | 74 | 162+ | 47,XX,inv(9)(p11q12)c,+21c |
| 63 | F/3 | 4.8 | 27 | 160+ | Missing |
| 64 | M/11 | 2.2 | 72 | 158+ | 47,X,-Y,+?der(9;21)(q10;p10),+21c,+mar |
| 65 | F/2 | 35.1 | 37 | 167+ | 48,XX,+2,+21c/49,idem,+X |
| 66 | M/4 | 6.5 | 127 | 95 | 47,XY,+21c |
| 67 | M/10 | 10.0 | 65 | 50 | 47,XY,+21c |
| 68^b^ | F/15 | 5.9 | NK | 155+ | 47,XX,del(3)(q13q13)x2,del(12)(q21q21),del(13)(q14q14),+21c [partly based on SNP array results] |
| 69 | F/4 | 17.7 | 52 | 145+ | 47,XX,+21c |
| 70 | M/12 | 41.9 | 49 | 147+ | Missing |
| 71^c^ | F/5 | 17.7 | 23 | 132+ | 48,XX,+X,del(1)(q41q44),add(7)(p?),del(9)(p11p22),+21c [partly based on BAC array results] |
| 72 | M/4 | 345 | 36 | 1 | 47,XY,add(19)(p13),+21c |
| 73 | F/6 | 3.6 | 359 | 33 | 47,XX,del(13)(q12),+21c |
| 74 | M/6 | 4.8 | 10 | 147+ | Missing |
| 75 | F/13 | 2.2 | 89 | 1 | Missing |
| 76 | M/1 | 28.8 | 15 | 122+ | 47,XY,+21c |
| 77 | M/6 | 4.3 | 57 | 131+ | 47,XY,del(12)(p13p13),+21c [partly based on FISH results] |
| 78 | F/6 | 5.8 | 120 | 130+ | 47,XX,+21c |
| 79 | M/2 | 8.6 | 19 | 41 | 49,XY,+X,+17,+21c |
| 80 | F/4 | 6.6 | 101 | 126+ | 49,XX,+X,+14,i(17)(q10),+21c |
| 81^b^ | M/5 | 123 | 72 | 108+ | 47,XY,der(4)t(X;4)(q24;p16),del(9)(p21p21),del(18)(p11),+21c [partly based on SNP array results] |
| 82^b^ | F/8 | 0.5 | 112 | 115+ | 56,XX,+X,del(1)(p11),dup(1)(q12q44),+4,+5,+6,dup(9)(p13p21),+11,+14,dup(17)(q21q25),+21c,+21,  +21,+21 [partly based on SNP array results] |
| 83 | M/16 | 5.7 | 41 | 119+ | 47,XY,t(14;14)(q11;q32),+21c |
| 84 | F/4 | 2.5 | 209 | 121+ | 57,XX,+X,+der(10),+13,+14,+14,+?15,+17,+18,+21c,+21,inc |
| 85 | F/3 | 36.0 | 64 | 113+ | 47,XX,?del(1)(q32),add(2)(q3?3),?del(5)(p13p14),+21c,add(21)(q22) |
| 86 | F/17 | 4.0 | 120 | 63+ | 47,XX,+21c |
| 87^b^ | M/4 | 71.0 | 11 | 106+ | 47,XY,del(1)(q42),del(12)(p12p13),del(12)(q21q21),dup(17)(q21q25),+21c [partly based on SNP  array results] |
| 88 | F/15 | 2.3 | 58 | 106+ | 47,XX,t(12;21)(p13;q22),+21c [partly based on RT-PCR results] |
| 89^b^ | M/8 | 65.0 | 19 | 33 | 47,dup(X)(q21q28),t(Y;14)(p11;q32),del(1)(p21p21),del(5)(q33q33),del(9)(p13p21),t(9;22)(q34;q11),  del(10)(p15p15),del(11)(q22),del(12)(q13q13),del(15)(q21q21),+21c [partly based on FISH and SNP  array results] |
| 90 | M/3 | 32.0 | 45 | 94+ | 47,XY,der(3)t(3;7)(q2?;p?),der(7)t(3;7)(?;p?),+21c,inc [partly based on FISH results] |
| 91 | F/3 | 7.9 | 126 | 88 | 47,XX,t(1;19)(q23;p13),?del(3)(p22p24),+21c |
| 92 | M/1 | 8.7 | 62 | 85+ | 47,XY,+21c |
| 93 | F/6 | 18.5 | 5 | 67 | 47,XX,t(12;21)(p13;q22),+21c [partly based on RT-PCR results] |
| 94^b^ | F/8 | 16.0 | 35 | 83+ | 48,XX,+X,del(9)(p21p21),+21c [partly based on SNP array results] |
| 95 | M/5 | 2.6 | 19 | 71+ | 47,XY,del(12)(p13p13),t(12;21)(p13;q22),+21c [partly based on FISH results] |
| 96 | F/15 | 10.3 | 114 | 80+ | 47,XX,t(12;21)(p13;q22),+21c [partly based on FISH results] |
| 97 | M/2 | 20.2 | 9 | 75+ | 47,XY,+21c |
| 98 | F/3 | 1.1 | 9 | 75+ | 47,XX,del(12)(p13p13),t(12;21)(p13;q22),+21c [partly based on FISH results] |
| 99 | M/4 | 198 | 41 | 1 | 47,XY,del(9)(p21),+21c |
| 100^b^ | M/13 | 27.0 | 27 | 1 | 47,del(X)(p11p11),Y,del(6)(p22p22),del(6)(q23q23),idic(7)(p11),del(10)(q25q25),del(12)(q21q21)x2,  +21c [partly based on SNP array results] |
| 101 | M/12 | 12.8 | 24 | 66+ | 47,XY,+21c |
| 102^b^ | M/14 | 13.8 | 11 | 21 | 47,XYc,t(X;14)(p22;q32),del(1)(q32q32),del(4)(q31q31),dic(7;16)(p11;p13),del(12)(q21q22),  del(13)(q14q14),del(16)(p12p12),dup(16)(p12p12),+21c [partly based on SNP array results] |
| 103^b^ | F/3 | 41.9 | 18 | 63+ | 47,X,del(X)(p22p22),+21c [partly based on SNP array results] |
| 104^b^ | M/12 | 16.1 | 76 | 64+ | 47,XY,del(3)(q26),del(12)(p12),t(12;21)(p13;q22),dup(18)(p11p11),dup(20)(p11p13),+21c  [partly based on FISH and SNP array results] |
| 105^b^ | F/11 | 10.1 | 7 | 10 | 49,XX,+del(3)(q26q26),del(4)(q31q31),del(5)(q14q14),del(5)(q31q32),+del(5)(q31),del(6)(q12q15),  t(6;20)(q13;q13),del(7)(p12p12),del(8)(q21q22),dup(8)(q22q24),idic(9)(p13),del(13)(q14q14),  der(19)t(1;19)(q21;p13),+21c [partly based on SNP array results] |
| 106 | F/4 | 127 | 12 | 61+ | 48-50,XX,del(1)(q41),+5,+21c,+21,inc |
| 107^c^ | F/14 | 94.1 | 25 | 2 | 47,XX,dup(8)(q21q24),del(13)(q13q34),dup(14)(q32q32),del(15)(q13q21),del(15)(q26q26),+21c  [partly based on BAC array results] |
| 108 | F/13 | 30.1 | 106 | 53+ | 47,XX,+21c |
| 109 | F/3 | 2.0 | 128 | 53+ | 46,XX,der(14;21)(q10;q10)c,+21c |
| 110 | M/6 | 0.8 | 83 | 59+ | 47,XY,+21c |
| 111 | M/6 | 15.0 | 13 | 53+ | 46,XY,der(5)t(4;5),der(16)t(5;16)/45,idem,der(16)t(15;21;16),-21/46,XY,der(21)t(15;21;16)c |
| 112 | M/2 | 91.0 | 17 | 49+ | Missing |
| 113 | M/7 | 0.6 | 24 | 47+ | 47,XY,del(12)(p13p13),t(12;21)(p13;q22),+21c [partly based on FISH results] |
| 114^b^ | M/5 | 7.0 | 142 | 12 | 49,XY,+X,del(5)(p15p15),del(9)(p21p21)x1-2,+21c,+21 [partly based on SNP array results] |
| 115 | F/5 | 22.5 | 13 | 1 | 47,XX,i(9)(q10),del(20)(q11),+21c |
| 116 | M/13 | 16.2 | 203 | 32+ | 48,XY,+X,+21c |
| 117 | M/1 | 4.9 | 34 | 32+ | 48,XY,+X,+21c |
| 118 | F/4 | 60.1 | 99 | 12 | 47,XX,+21c |
| 119 | M/5 | 49.4 | 114 | 35+ | 47,XY,+21c |
| 120^b^ | F/3 | 2.8 | 147 | 27+ | 47,XX,+21c |
| 121 | M/4 | 4.5 | 75 | 30+ | 47,XY,+21c |
| 122 | F/16 | 27.8 | 74 | 19 | 47,XX,+21c |
| 123 | M/4 | 5.8 | 11 | 24+ | 47,XY,+21c |
| 124 | F/3 | 17.4 | 17 | 17+ | 47,XX,i(9)(q10),+21c |
| 125 | M/3 | 2.5 | 30 | 14+ | 47,XY,+21c |
| 126^b^ | F/13 | 7.5 | 30 | 19+ | 47,XX,del(3)(q13q13),del(13)(q14q14)x2,+21c [partly based on SNP array results] |
| 127 | F/3 | 39.0 | 5 | 15+ | Missing |
| 128 | F/1 | 117 | 8 | 15+ | 48,XX,+10,+21c |

BAC, bacterial artificial chromosome; DS-ALL, Down syndrome-related acute lymphoblastic leukemia; F, female; FISH, fluorescence *in situ* hybridization; M, male; NK, not known; Plts, platelet count; RT-PCR, reverse-transcription polymerase chain reaction; SNP, single nucleotide polymorphism; WBC, white blood cell count; +, alive at the time of reporting.

^a^Cases listed in chronological order from 1981 until 2010.

^b^Karyotypes have previously been published by Lundin *et al* (2012).

^c^Karyotypes have previously been published by Lundin *et al* (2009).

**References**

Lundin, C., Heldrup, J., Ahlgren, T., Olofsson, T. & Johansson B. (2009) B-cell precursor t(8;14)(q11;q32)-positive acute lymphoblastic leukemia in children is strongly associated with Down syndrome or with a concomitant Philadelphia chromosome. *European Journal of Haematology,* **82,** 46-53.

Lundin, C., Hjorth, L., Behrendtz, M., Ehinger, M., Biloglav, A. & Johansson B. (2012) Submicroscopic genomic imbalances in Burkitt lymphomas/leukemias: association with age and further evidence that 8q24*/MYC* translocations are not sufficient for leukemogenesis. *Genes Chromosomes & Cancer*, **52**, 370-377.
